# A unified multi‐activation (UMA) model of cell survival curves over the entire dose range for calculating equivalent doses in stereotactic body radiation therapy (SBRT), high dose rate brachytherapy (HDRB), and stereotactic radiosurgery (SRS)

**DOI:** 10.1002/mp.14690

**Published:** 2021-02-16

**Authors:** Shidong Li, Curtis Miyamoto, Bin Wang, Tawfik Giaddui, Bizhan Micaily, Andrew Hollander, Stephanie E. Weiss, Michael Weaver

**Affiliations:** ^1^ Department of Radiation Oncology Temple University Hospital Philadelphia PA USA; ^2^ Department of Radiation Oncology Fox Chase Cancer Center Temple University Health System Philadelphia PA USA; ^3^ Department of Neurosurgery Temple University Health System Philadelphia PA USA

**Keywords:** EQD2, HDR, Radiobiological Outcome modeling, SBRT, SRS

## Abstract

**Purpose:**

Application of linear‐quadratic (LQ) model to large fractional dose treatments is inconsistent with observed cell survival curves having a straight portion at high doses. We have proposed a unified multi‐activation (UMA) model to fit cell survival curves over the entire dose range that allows us to calculate EQD2 for hypofractionated SBRT, SRT, SRS, and HDRB.

**Methods:**

A unified formula of cell survival $S=n/\left(e^{\frac{D}{D_{o}}}+n‐1\right)$ using only the extrapolation number of n and the dose slope of *D_o_* was derived. Coefficient of determination, R^2^, relative residuals, r, and relative experimental errors, e, normalized to survival fraction at each dose point, were calculated to quantify the goodness in modeling of a survival curve. Analytical solutions for α and β, the coefficients respectively describe the linear and quadratic parts of the survival curve, as well as the α/β ratio for the LQ model and EQD2 at any fractional doses were derived for tumor cells undertaking any fractionated radiation therapy.

**Results:**

Our proposed model fits survival curves of in‐vivo and in‐vitro tumor cells with R^2^ > 0.97 and r < e. The predicted α, β, and α/β ratio are significantly different from their values in the LQ model. Average EQD2 of 20‐Gy SRS of glioblastomas and melanomas metastatic to the brain, 10‐Gy × 5 SBRT of the lung cancer, and 7‐Gy × 5 HDRB of endometrial and cervical carcinomas are 36.7 (24.3–48.5), 114.1 (86.6–173.1),, and 45.5 (35–52.6) Gy, different from the LQ model estimates of 50.0, 90.0, and 49.6 Gy, respectively.

**Conclusion:**

Our UMA model validated through many tumor cell lines can fit cell survival curves over the entire dose range within their experimental errors. The unified formula theoretically indicates a common mechanism of cell inactivation and can estimate EQD2 at all dose levels.

## 1 INTRODUCTION

Linear‐quadratic model (LQ) that can fit the initial shoulder of cell survival curves at the low‐dose domain has been extended for calculation of equivalent dose in 2‐Gy fractions (EQD2) to the tumor and organs at risk (OARs) receiving large fractional doses during hypofractionated stereotactic body radiation therapy (SBRT), high dose rate brachytherapy (HDRB), and intracranial stereotactic radiation therapy (SRT) or stereotactic radiosurgery (SRS). Such an extrapolation by using the same α/β ratio derived at 2‐Gy fractions might be inappropriate to predict cell survival at 7–20‐Gy fractions, which are most likely located within the straight or almost straight portion of observed cell survival curves.^1^, ^2^

Correct estimation of radiation responses of human tumors and organs at risk (OARs) based on the intrinsic radiosensitivity of the cell lines measured in‐vivo or in‐vitro is required in design of any radiation therapy schemes particularly for hypofractionated radiation therapy with a high dose per fraction. Gurrero and Li^2^ have extended the LQ model pertinent to stereotactic radiotherapy by modifying the β parameter into a product of the β and a function of time and repairing‐rate constants. This modified LQ model has improved the fitting of cell survival curves at high doses as does by a four‐parameter‐based lethal and potential lethal (LPL) damage repairing model.^3^, ^4^, ^5^ However, models with more parameters and complicated functions are more difficult to use than the simple LQ and multitarget (MT) models each using only two parameters. Garcia *et al*.^6^ have derived the α and β parameters by fitting cell survival curves for different dose ranges. However, a prediction from such a model is restricted to the individual dose ranges. Park *et al*.^7^ have proposed a universal survival curve (USC) model to calculate equivalent dose by applying an LQ model at the low‐dose domain, a MT model at the high‐dose domain, and an analytical formula for determination of the transition dose point between the two dose domains. Astrahan^8^ has then added a linear tail function at the low dose domain and Kehwar *et al*.^9^ have modified the transition dose formula using D_o_, n, and α to improve the USC model. A review of the radiobiology for SBRT by Garau *et al*.^10^ has outlined some differences between the LQ and USC models in the calculation of BED and EQD2. More importantly, application of the USC uses different descriptions of radiosensitivity of tumor or tissue cells when their fractional doses change across the transition dose during SBRT, SRT, and HDRB. In addition, there is no evidence of cell responses suddenly switching at the “transition dose point”. A better understanding of cell responses to avoid a systematic discrepancy between any model predictions and experimental observations at all dose levels is urgently needed for evaluation of hypofractionated radiotherapy^11^, ^12^ in light of recent technological advances.

This article attempts to resolve the radiobiological discrepancies (or catastrophes) between observed survival curves and model predictions, at either high‐dose domain by the LQ model or at low‐dose domain by the MT model, by introducing a UMA model to fit all cell survival curves over the entire dose range. A unified formula using only two parameters is empirically derived and then numerically validated with many observed survival curves that we could find and redraw from published data that have either repeated measurements or estimated experimental errors. Such a formula using dose‐independent parameters allows us to estimate EQD2 and the α and β parameters for the LQ model at any doses per fraction.

## 2 METHODS AND MATERIALS

### 2.1 A UMA Model derived from an observed cell survival curve

**Fig. 1:**
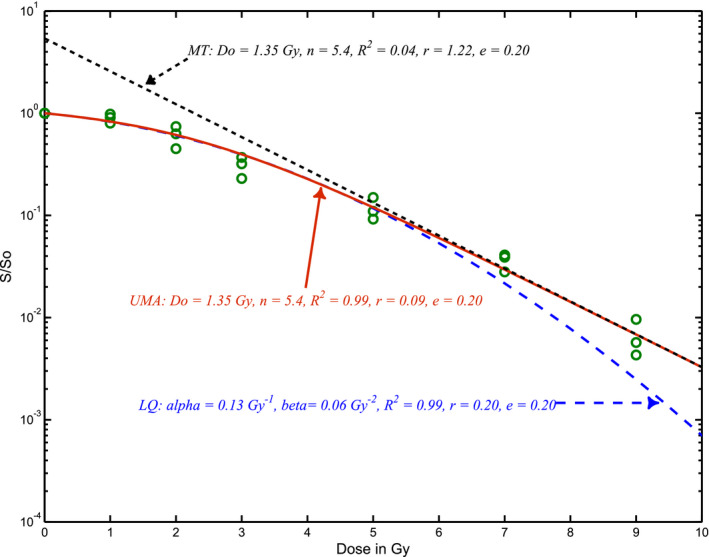
Comparison of the MT, UMA and LQ modeling of an x‐ray survival curve of MelH — a human melanoma cell line with triplet measurements redrawn from Weichselbaum RR *et al*.^13^ The S/S_o_ = 1 at the zero dose is included.

Figure 1 illustrates three repeatedly measured x‐ray survival fractions of a human melanoma cell line by Weichselbaum *et al*.^13^ which were refit by the MT model using the linear least square regression of the natural logarithm of the cell survival fraction $S=ne^{‐D/D_{o}}$ for doses > 3 Gy with $D_{o}$= 1.35 Gy and n = 5.4. The MT model overestimated cell survival at the initial shoulder (low doses) that results a poor R^2^ = 0.04 and a large residual r = 1.22. The initial shoulder was better fit by the second‐order polynomial trend line of the natural logarithm of the LQ model^14^ $S=e^{‐\alpha D‐\beta D^{2}}$ for the low dose data with results of α = 0.13 Gy^−1^ and β = 0.06 Gy^−2^, respectively. However, the LQ model underestimated the survival at high doses ≥ 7 Gy. The first author has proposed a cell survival formula of $S=a/\left(e^{\mathrm{bD}}+c\right)$, where a, b, and c are dose‐independent parameters. We now determine the formula through an empirical approach with no need of a theoretical derivation to be presented in another report. Prior to irradiation at dose of $D=0\;\text{Gy},\;\text{we have S}=1=\frac{a}{e^{\mathrm{bD}}+c}=\frac{a}{1+c},\;\text{thus},a=1+c$. At high doses with $e^{\mathrm{bD}}$ >> c, the survival curve is almost straight as $S\approx ne^{‐\frac{D}{\mathrm{Do}}}=a/e^{\mathrm{bD}}$ so that *a* = n, *b* = 1/*D_o_* and c=n‐1. Now, we have obtained a unified formula of the survival curve using only two parameters as:

(1) $$S=n/\left(e^{\frac{D}{D_{o}}}+n‐1\right).$$

The parameters of n and $D_{o}$ are similar to that of the MT model but iteratively determined through first setting of $D_{o}=‐\frac{D_{i}‐D_{i‐1}}{\text{ln}\left(S_{i}\right)‐\text{ln}\left(S_{i‐1}\right)}\text{and}\;n=\;S_{i‐1}e^{D_{i‐1}/D_{o}}$ at the two high dose points of *D_i_* and *D_i‐1_*, then adjusting the n and *D_o_* with the linear regression using least squares of Ln(*n/S_i_ + *1*‐n*) verses *D_i_/D_o_* at all measured dose points for having the lowest relative residual (r) of modeled responses of *S*(*D_i_*) from the observed S_i_ as $r=\frac{1}{I}\sum_{i=1}^{i=I}S\left(D_{i}\right)‐S_{i}/S_{i}$ while maintaining a good coefficient of determination of $R^{2}=1‐\frac{\mathrm{RSS}}{\mathrm{SSS}}$, where $RSS=\sum_{i=1}^{i=I}{\left(S\left(D_{i}\right)‐S_{i}\right)}^{2}$ and $SSS=\sum_{i=1}^{i=I}{\left(S_{i}‐\langle S\rangle \right)}^{2}$ are the residual sum of squares and the corrected sum of squares of survival^15^, respectively. The relative residual of *r* is compared with the relative experimental errors of e = $\frac{1}{I}\sum_{i=1}^{i=I}\Delta S_{i}/S_{i}$, where $\Delta S_{i}$ is one standard deviation of the multiple survival measurements at a dose point of *i*. ***I*** is the total number of dose points in the survival curve. The relative residuals are the second index of good fitness in addition to the R^2^ since R^2^ is insensitive to the discrepancy at the high doses with very low survival fractions as shown in Fig. 1 between the LQ and UMA models sharing the same R^2^ but different r. Typically, a good fitting of a survival curve should have R^2^ in a range of 0.9–1.00 and the relative residual smaller than that of the relative experimental errors, that is, r < e. In congruence with survival curves in literatures, mean values and one standard deviations of the survival fractions at individual dose points are redrawn and used in our curve fitting. Survival fraction normalized to 1 with no error bar at the zero dose is used for all CSCs.

### 2.2 Analytical Predictions of EQD2 for any radiotherapy schemes

There is only a single term of $e^{D/D_{o}}$ that contains the independent variable of dose D in Eq. (1) for modeling the cell survival, thus, the biologically effective dose (BED) can be the physical dose D and there is no need for BED calculation in our UMA model. To achieve the same ending survival *S*end by a course of D‐Gy N fractions as that treated with 2‐Gy N_2_ fractions, that is, $S_{\mathrm{end}}=S{\left(D\right)}^{N}=S{\left(2Gy\right)}^{N_{2}}$, we have the $N_{2}=N\frac{Ln\left(n\right)‐Ln\left(e^{\frac{D}{D_{o}}}+n‐1\right)}{Ln\left(n\right)‐Ln\left(e^{\frac{2Gy}{D_{o}}}+n‐1\right)}$ and more importantly:

(2) $$EQD2=2Gy\cdot N\frac{Ln\left(n\right)‐Ln\left(e^{\frac{D}{D_{o}}}+n‐1\right)}{Ln\left(n\right)‐Ln\left(e^{\frac{2Gy}{D_{o}}}+n‐1\right)}.$$

Since n and D_o_ are independent of the fractional dose of D, Eq. (2) allows us to calculate the EQD2 of the tumor or tissue cells receiving any fractional doses. If D changes with different sessions, the EQD2 at the end of treatment is just the summation of individual fractional EQD2. Another advantage of the proposed UMA model is to deal with tumor cells under hypoxic condition by simply changing the D_o_ and n for the hypoxic tumor cells in Eq. (2).

The LQ model has also assumed $S_{\mathrm{end}}=S{\left(D\right)}^{N}=e^{‐\alpha ND‐\beta ND^{2}}=e^{‐\alpha BED}$ with $BED=ND\left(1+D/\left(\alpha /\beta \right)\right)=D_{\mathrm{total}}\left(1+D/\left(\alpha /\beta \right)\right)$ so that Ln(S) (the natural logarithm of survival fraction) will linearly correlate with the BED that compose factors of the total physical dose $D_{\mathrm{total}}$ and $\left(1+D/\left(\alpha /\beta \right)\right).$ To achieve the same ending survival (or BED if there is no change of α with the fractional dose) as that of 2‐Gy fractions, one could determine the equivalent dose in 2‐Gy per fraction: $EQD2=ND\left(1+D/\left(\alpha /\beta \right)\right)/\left(1+2Gy/\left(\alpha /\beta \right)\right)$. The EQD2 estimated by the LQ model would have the same value or dose as Eq. (2) if both the α and α/β ratio in the LQ model were dose independent.

### 2.3 α and α/β ratio varied with D

LQ model defines $\text{Ln}\left(S\right)=‐\alpha D‐\beta D^{2}$ and by replacing S with our Eq. (1), we can determine the α and β parameters by the first and second derivatives of Ln(S) as:

(3) $$\beta =‐\frac{\partial^{2}\left(\text{Ln}\left(S\right)\right)}{2\partial D^{2}}=\frac{\partial^{2}Ln\left(e^{\frac{D}{D_{0}}}+n‐1\right)}{2\partial D^{2}}=\frac{\left(n‐1\right)e^{D/D_{o}}}{2D_{o}^{2}{\left(e^{\frac{D}{D_{0}}}+n‐1\right)}^{2}}$$

(4) $$\alpha =‐\frac{\partial Ln\left(e^{\frac{D}{D_{0}}}+n‐1\right)}{\partial D}+2\beta \cdot D=\frac{e^{D/D_{o}}}{D_{o}\left(e^{\frac{D}{D_{0}}}+n‐1\right)}+\frac{D\left(n‐1\right)e^{D/D_{o}}}{D_{o}^{2}{\left(e^{\frac{D}{D_{0}}}+n‐1\right)}^{2}}\;\text{and}$$

(5) $$\alpha /\beta =\frac{2D_{o}\left(e^{\frac{D}{D_{0}}}+n‐1\right)}{n‐1}‐2D.$$

Clearly, α, β and α/β ratio change significantly with the fractional dose of *D* and our UMA determines the EQD2 by Eq. (2) without using the dose dependent α, β and α/β ratio.

### 2.4 Interpretation of the new UMA model

There are a number of radiobiological models and the choice of a model particularly for the prediction with extrapolations from low dose of ~2 Gy to high doses of ~10 Gy has been a major concern in clinical implementation.^16^ The proposed UMA model is conceptually different from other models (including the latest USC model) by fitting both the shoulder and straight portions without any mechanism changes. In fact, recent studies have demonstrated that radiation induced DNA double‐strand breaks (DSB) have the same nonhomologous end‐joining (NHEJ) as the mainstay for sublethal damage repairing (SLDR) after a low‐dose fraction and potentially lethal damage repairing (PLDR) after a high‐dose fraction.^17^, ^18^ If both SLDR and PLDR depend mostly on NHEJ of DSB of DNA, it is reasonable to explore the UMA model for a simple and fundamental quantification of radiosensitivity of cells. For which, we have adapted the mean inactivation dose (MID) previously introduced for fractionated radiotherapy and brachytherapy by Fertil *et al*.^19^ and we have obtained an analytical solution of

(6) $$MID=\int_{0}^{\infty }S\left(D\right)dD=n\int_{0}^{\infty }\frac{\mathrm{dD}}{e^{\frac{D}{D_{o}}}+n‐1}=n{\left(\left.\frac{D‐D_{o}Ln\left(e^{\frac{D}{D_{o}}}+n‐1\right)}{\left(n‐1\right)}\right|\right)}_{0}^{\infty }=\frac{nLn\left(n\right)D_{o}}{n‐1}.$$

**Fig. 2:**
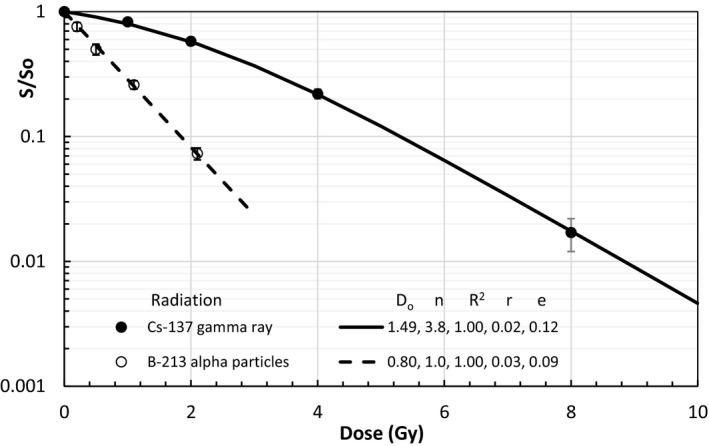
Modeling of MDA‐MB‐231cells — a human breast cancer, irradiated with Cs‐137 γ‐ rays or Bi‐213 α‐ particles redrawn data from Fig. 4 of Hobbs, RF *et al*.^20^.

The MID is determined by the D_o_ and n of the cells receiving the radiation. One can compare the MID with the mean life span of a radioactive source which is an integration of “radioactive nuclei or excited atoms” over the time from 0 to ∞ with a result of T = 1/λ = 1.44 T_1/2_. Where λ and T_1/2_ are decay constant and half‐life, respectively. The life span T is a single parameter to describe the activity change with time and the MID may be used as the single parameter to describe the cell response to the radiation dose. Certainly, MID is more complicated than the life span and the n may serve as the number of activated cell death pathways of a live cell by the radiation dose. In fact, if n = 1, the survival curve is exactly given by $S=e^{‐D/D_{o}}$ and the *MID* = *D_o_*. The number of active pathways, n, may change with the type of radiation as shown by Fig. 2 for the change of D_o_ from 0.80 to 1.49 Gy and n from 1.0 to 3.8 to the same breast cancer cell line irradiated with α particles to γ‐rays, respectively. Eq. (6) tells us that the change of MID of the breast cancer from 0.80 Gy for α‐particle irradiation to 2.7 Gy for the γ‐ray irradiation is magnified by the changes of D_o_ and n. Thus, the activation number n plays an important role in MID or cell killings from the ionization radiation. We have named the new model as the unified multi‐activation (UMA) model instead of unified multi‐target model since the cell survival corresponds to the inactivation of n‐activated pathways by the specific radiation and our modeled n and D_o_ are differently from that of the MT model.

## 3 RESULTS

### 3.1 Survival curves of human cells fitted with the UMA model

**Fig. 3:**
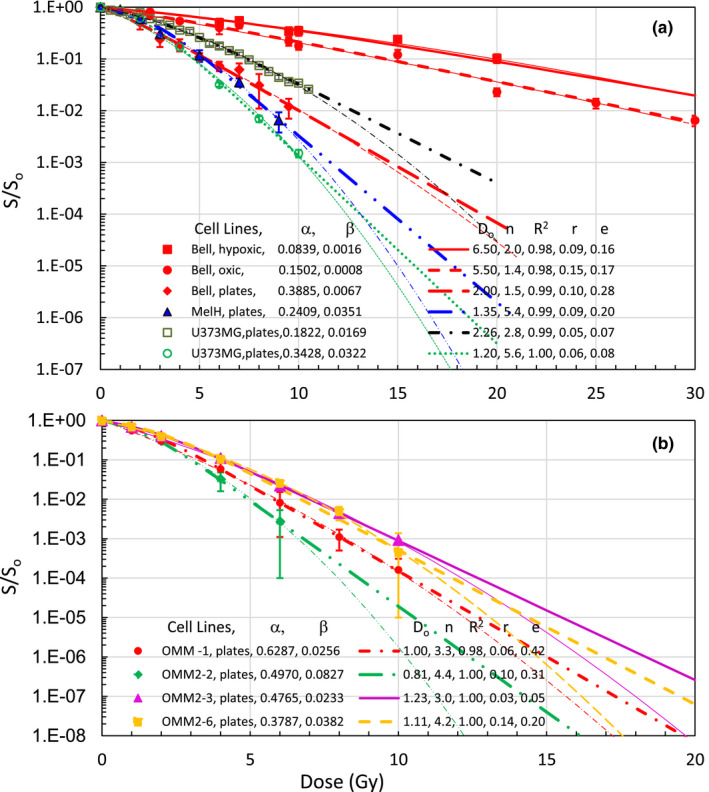
(a) Survival curves of human melanomas: in vivo and in vitro Bell cells from Guichard *et al*.^22^ and MelH^13^, human glioblastomas: U373MG (in open square) from Raaphorst GP *et al*.^23^ and (in open circle) from Garcia LM *et al*.^6^. D_o_ of in‐vivo Bell cells is much higher than that of in vitro. (b) Four human uveal melanomas metastatic to the brain and liver: OMM‐1, OMM 2‐2, OMM2‐3, and OMM2‐6 redrawn from van den Aardweg *et al*.^24^. Thin curves are LQ model fitting for all dose points in individual curves. Both LQ and UMA models have fitted individual survival curves well with R^2^ > 0.98 and r < e, but there are systematic differences in high doses (>30 Gy to show the difference for in‐vivo Bell cells).

Our UMA model has provided the best fit among the LQ, MT and UMA models for in‐vivo and in‐vitro cell lines we have found in literature search with presentation of error bars or repeated measurements as shown in Fig. 1 with three measurements on individual dose points. The UMA model has also the best fit of three skin fibroblast cell lines from courtesy of Weichselbaum *et al*.^21^ (not presented here). Survival curves of some malignant tumor cell lines of interest in intracranial SRS are presented in Fig. 3(a) for in‐vivo hypoxic and oxic as well as in‐vitro plated Bell cells ^22^ and plated MelH cells^13^ — two metastatic melanoma lines and two plated U373MG cells — a glioblastoma line ^6^, ^23^ and in Fig. 3(b) for four metastatic uveal melanomas of OMM‐1, OMM 2‐2, OMM2‐3, and OMM2‐6 cells.^24^ Apparently, in‐vivo hypoxic and aerobic Bell cells have high D_o_ of 6.5 and 5. 5 Gy, respectively. The extrapolation number n varies significantly with the cell lines. All thin lines are LQ model fitting curves with R^2^ > 0.98 but systematically differing from thick lines of UMA model curves at high doses. Our α/β ratios should agree with that of the original publications but results of 24.6 and 20.5 Gy for the OMM‐1 and OMM 2‐3 cell lines are respectively doubled the original values of 14.1 ± 2.6 and 10.3 ± 1.6 Gy ^24^ which is due to the exclusion of the highest dose point for the LQ model fitting of the curves in the original report. Thus, α/β ratio of LQ is extremely sensitive to the dose range.

**Fig. 4:**
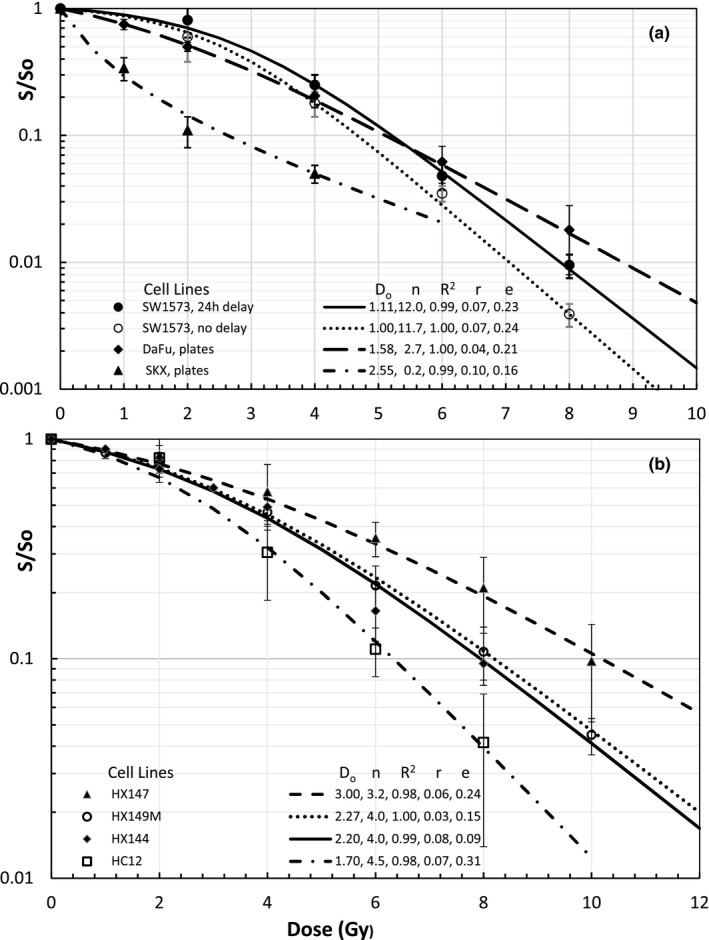
UMA modeling of survival curves of tumors for SBRT: 4(a) human lung squamous cell cancer (SCC) — SW1573 cells with 24‐hour delay plates and immediate plates after irradiation redrawn data from Franken NAP *et al*.^25^. In vitro data of DaFu Cells — a H&N SCC and SKX — a base of tongue SCC redrawn from Menegakis A *et al*.^26^. The inverted shoulder of SKX cells has a good fitting with D_o_ = 2.55 Gy and n = 0.2; 4(b) In vivo lung cancer other than SCC redrawn data from Duchesne GM *et al*.^27^ for HX147 — a large‐cell carcinoma, HX149M — a variant small cell carcinoma, HX144 — an adenocarcinoma, and HC12 — a classical small cell lung cancer (SCLC).

Results of tumors treated with extracranial SBRT are grouped in Fig. 4(a) for survival curves of squamous cell cancer (SCC) cell lines: 24‐hour delay and no delay plated SW1573 — a lung SCC,^25^ DaFu — a head and neck SCC ^26^ and SKX — a base of tongue SCC^26^ and in Fig. 4(b) for in‐vivo lung cancer lines other than SCC including HX147 — large‐cell carcinomas, HX149M — variant small cell carcinomas, HX144 ‐Adenocarcinomas, and HC12 — classical small cell lung cancer (SCLC).^27^ The invert shoulder having a negative curvature of SKX cells in Fig. 4(a) was fitted well with D_o_ = 2.55 Gy and n = 0.2.

**Fig. 5:**
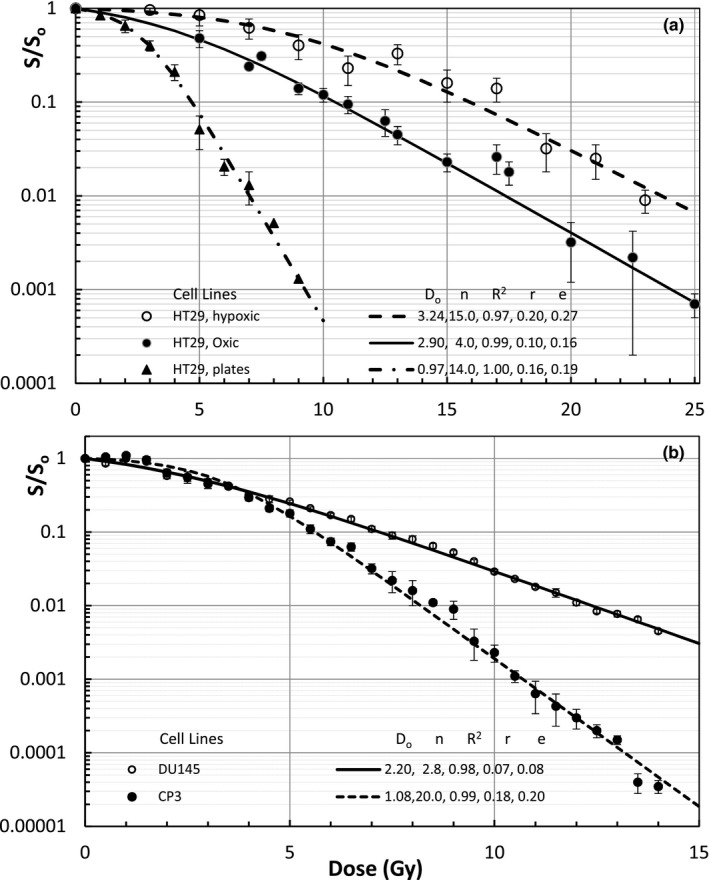
(a) Modeling of in vivo and in vitro survival curves of a colorectal cancer cell line of HT29 redrawn data from Guichard M *et al*.^22^; 5(b) In vitro prostate cell lines of DU145 and CP3 redrawn data from Garcia LM *et al*.^6^. All survival curves fitted by the UMA model have R*^2^* > 0.97 and r < e with different D_o_ and n.

Figure 5(a) represents in‐vivo and in‐vitro survival curves of HT29 cells — a colorectal cancer cell line, under hypoxic, aerobic, and no delay plate conditions.^22^ Fig. 5(b) shows two prostate cell lines of DU145 and CP3 cells ^6^ with significantly different D_o_ = 2.20 and 1.08 Gy and n = 2.8 and 20.0, respectively.

**Fig. 6:**
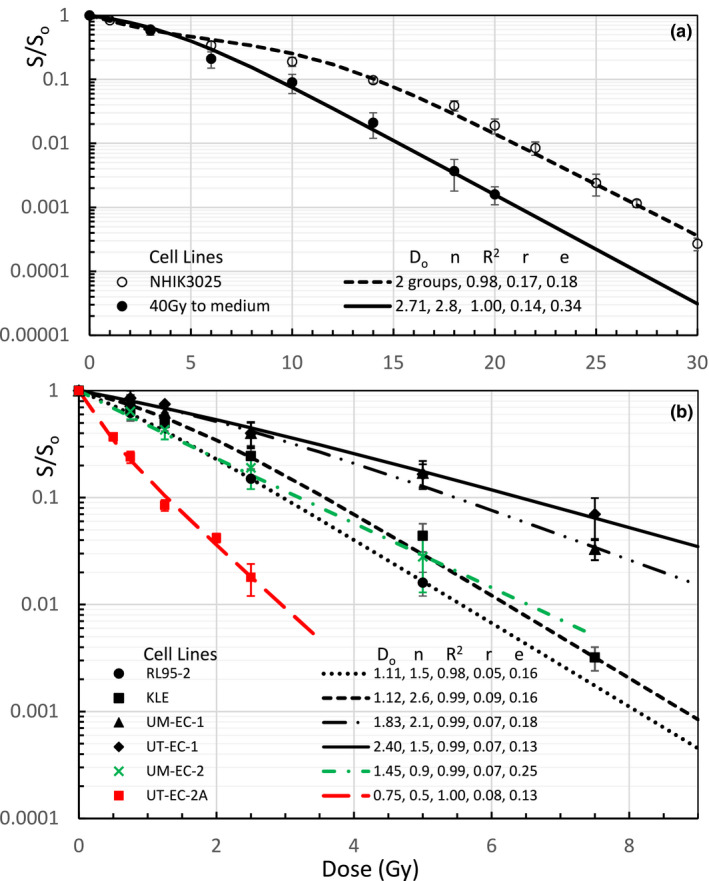
(a) Survival Curves of a cervical adenocarcinoma, NHIK 3025 cells, irradiated under extreme hypoxia for a contact medium with no or 40 Gy preradiation redrawn from Figs. 1 and 2 of Pettersen EO *et al*.^28^ with an addition of survival fraction of 1 at zero dose. The irregular shoulder for the cells with no pre‐irradiation were fitted with two groups of cells: one half with D_o_ = 2.80 Gy and n = 1.0 and the other half with D_o_ = 2.70 Gy and n = 58 for the entire dose range; (b) In vitro survival curves of endometrial carcinomas and their metastatic cell lines (6 out 22 curves from Figs. 1A‐I of Rantanen V *et al*.^29^ presented). The metastatic endometrial carcinomas of UM‐EC‐2 and UT‐EC‐2 cells had n < 1. All UMA modeled curves had R^2^ > 0.98 and r < e.

Figures 6(a) and 6(b) address the tumor cell lines for HDRB with some irregular survival curves of the cervical adenocarcinomas irradiated under extreme hypoxia with or without pre‐irradiation of the contact medium^28^ and some in‐vitro survival curves of the endometrial carcinomas,^29^ respectively. For all of the tested cell lines, our new model has R^2^ > 0.97 and relative residuals r < e. In other words, the UMA model has described all cell survival curves within the experimental uncertainties. The survival curves of a SKX cell line in Fig. 4(a) and two metastatic endometrial carcinomas (EC) of UM‐EC‐2 and UT‐EC‐2 in Fig. 6(b) have negative curvatures and they are all well fitted with n < 1. The irregular survival curves of NHIK 3025 Cells in Fig. 6(a) could be fitted by two cell populations (or groups): one half with D_o_ = 2.80 Gy and n = 1 and the other half with D_o_ = 2.70 Gy and n = 58 using the same formula of Eq. (1) over the entire dose range. The only curve with R^2^ = 0.97 in Fig. 5(a) for HT29 Hypoxic cells could also be improved by using two populations. The UMA modeling over the entire dose range is fundamentally different from fitting the survival curves with separated dose ranges.^6^, ^7^

### 3.2 α, α/β and EDQ2 Determined with the UMA Model

Table I has listed UMA modeled parameters of D_o_, n and calculated α, α/β ratio and EQD2 for some fractional doses other than 2 Gy. The last column lists the EDQ2 from commonly adopted LQ model using α/β = 3 Gy for the prostate cancer, 8 Gy for the lung and colorectal cancer, and 10 Gy for all other tumors based on clinical studies.^30^, ^31^ Using Eq. (4), our model predicted α values from 0.17 to 0.62 Gy^−1^ for in‐vivo cells and from 0.44 to 1.3 Gy^−1^ for in‐vitro cells at these specified fractional doses are mostly greater than the α values from 0.02 to 0.3 Gy^−1^ derived for the fractional dose at 2 Gy by the LQ model.^30^ Using Eq. (5), α/β ratios determined with our model at fractional doses ≥ 7 Gy are mostly orders of magnitude higher than the typical values of ≤10 Gy for current LQ model derived from clinical data receiving a fractional dose of 2 Gy. Some larger α/β values about 20 Gy have been determined by Maciejewski *et al*.^32^ from tumor control probabilities (TCP) observed on 498 squamous cell carcinomas of the oral cavity and oropharynx treated with the fractional doses ranging from 1.7 to 4.5 Gy and a recent study by Liu *et al*.^33^ from 1, 2, 3 and 5 yr TCP data of 46 selected studies of SBRT of early stage nonsmall cell lung cancer using the standard LQ and five improved LQ models for a group of 211 patients receiving fractional doses from 3 to 4 Gy and a group of 3268 patients receiving fractional doses >6 Gy. The low fractional dose group^33^ has the dose points dominated in the exponential and maturity phases of the sigmoid shape of TCP curves and they are influential points with high leverage in determination of their α/β ratios. In fact, our calculated α/β ratios of 19 to 38 Gy for colorectal adenocarcinomas at a fractional dose of 5.0 Gy and 22 Gy for a Head and Neck squamous cell carcinoma cell line at a fractional dose of 3.0 Gy are consistent with the newly derived α/β values.

**Table I:** UMA modeled D_o_, n and predicted α, α/β and EQD2 of Human tumors for D‐Gray N‐fractions

Cell Lines	Tumors	Ref.	D_o_ (Gy)	n	D (Gy)	N	α (Gy^−1^)	α/β (Gy)	EQD2 (Gy)	EDQ2 LQ(Gy)
Hypoxic Bell	Melanoma	22	6.50	2.0	20.0	1	1.7E‐01	3.3E + 02	29.3	50.0
Oxic Bell			5.50	1.4	20.0	1	1.9E‐01	1.1E + 03	24.3	50.0
Bell,MelH		13,22	1.68	3.5	20.0	1	6.0E‐01	2.1E + 05	41.9	50.0
OMM‐1	Metastatic Melanoma	24	1.00	3.3	20.0	1	1.0E + 00	4.2E + 08	34.9	50.0
OMM 2‐2			0.81	4.4	20.0	1	1.2E + 00	2.5E + 10	37.4	50.0
OMM 2‐3			1.23	3.0	20.0	1	8.1E‐01	1.4E + 07	35.3	50.0
OMM 2‐6			1.11	4.2	20.0	1	9.0E‐01	4.6E + 07	41.9	50.0
U373MG	Glioblastoma	6,23	1.73	4.2	20.0	1	5.8E‐01	1.1E + 05	48.5	50.0
SW1573	Lung SCC	25	1.00	11.7	10.0	5	1.0E + 00	4.1E + 03	173.1	90.0
In‐vivo HX147	LCLC	27	3.00	3.2	10.0	5	3.8E‐01	1.0E + 02	86.6	90.0
In‐vivo HX149M	Variant SCLC		2.27	4.0	10.0	5	4.9E‐01	1.5E + 02	101.0	90.0
in vivo HX144	Lung AdC		2.20	4.0	10.0	5	5.0E‐01	1.6E + 02	101.2	90.0
in vivo HC12	class SCLC		1.70	4.5	10.0	5	6.2E‐01	3.7E + 02	108.5	90.0
NHIK 3025	Cervical AdC	28	2.80	1.0	7.0	5	3.6E‐01	N/A	35.0	49.6
			2.70	58.0	7.0	5	2.2E‐01	2.1E + 01	103.1	49.6
	40Gy Med		2.71	2.8	7.0	5	4.3E‐01	5.9E + 01	51.1	49.6
RL95‐2	Primary EC	29	1.11	1.5	7.0	5	9.1E‐01	2.4E + 03	40.0	49.6
KLE			1.12	2.6	7.0	5	9.1E‐01	7.4E + 02	49.6	49.6
UM‐EC‐1			1.83	2.1	7.0	5	5.8E‐01	1.7E + 02	46.7	49.6
UT‐EC‐1			2.40	1.5	7.0	5	4.4E‐01	2.0E + 02	40.6	49.6
UM‐EC‐2	Metastatic EC		1.45	0.9	1.2	40	6.9E‐01	‐6.1E + 01	48.7	52.8
UT‐EC‐2			0.75	0.5	1.2	40	1.2E + 00	‐1.1E + 01	52.6	52.8
SKX	BOT SCC	26	2.55	0.2	1.2	60	4.2E‐01	‐2.7E + 00	85.8	79.2
DaFu	H&N SCC		1.58	2.7	3.0	18	7.0E‐01	2.2E + 01	61.4	47.3
Hypoxic HT29	Colorectal AdC	22	3.24	15.0	5.0	5	1.7E‐01	1.9E + 01	39.6	32.5
Oxic HT29			2.90	4.0	5.0	5	3.6E‐01	2.7E + 01	34.6	32.5
Plates HT29			0.97	14.0	5.0	5	1.3E + 00	3.8E + 01	64.9	32.5
DU145	Prostate AdC	6	2.20	2.8	7.4	5	5.1E‐01	9.0E + 01	56.4	77.0
CP3			1.08	20.0	7.4	5	1.0E + 00	1.2E + 02	162.9	77.0
MDA‐MB‐231	γ‐rays, BC	20	1.49	3.8	2.66	16	7.2E‐01	1.5E + 01	48.1	44.9
	α‐particles, BC		0.80	1.0	2.50	5	1.3E + 00	N/A	56.2^a^	13.0

Abbreviations: AdC = adenocarcinoma; BC = Breast Cancer; BOT = the base of tongue; EC = endometrial carcinoma; H&N = the head and neck; LCLC = large cell lung cancer; SCC = squamous cell carcinoma; SCLC = small cell lung cancer.

^a^ EQD2 for α‐particle irradiation is determined by using α‐particle parameters in the numerator and γ‐ray parameters in the denominator of Eq. (2).

A big advantage of UMA model is that the parameters of n and D_o_ can be determined from preclinical measurements of cell survival curves within experimental dose ranges while α and α/β ratios determined with LQ models change significantly with dose ranges. There are negative α/β ratios for the tumors with a negative curvature (β < 0 and n < 1) for which hyper‐fractionated 1.2‐Gy twice‐daily radiation (BID) irradiation is desired. EQD2 for 20 Gy SRS of the primary glioblastomas or metastatic melanomas, range from 24.3 to 48.5 Gy with an average of 36.7 Gy, much lower than 50 Gy from the LQ model. EQD2 of 10 Gy × 5 SBRT of the lung carcinomas, except for the large cell lung carcinomas with 86.6 Gy, ranging from 101 to 173 Gy are much higher than 90 Gy from the LQ model (EQD2 = 83.3 Gy if α/β = 10 Gy). EQD2 for 3 Gy × 18 of H&N SCC, 5 Gy × 5 of colorectal adenocarcinomas and 2.66 Gy × 16 of the breast cancer are all higher than that from the LQ model. EQD2 for 7 Gy × 5 HDRB of cervical and endometrial carcinomas, except for the mixed cell population survival curve of NHIK3025, varies from 40 to 52.6 Gy. EQD2 for any tumors with mixed cell populations is calculated by using the low EQD2 of the most radioresistant cell population since the tumor cells would be dominated by the radioresistant cells at the end of treatment. For the case of 7 Gy × 5 HDRB of cervical cancer of a NHIK survival curve with n = 1 and D_o_ = 2.8 Gy, UMA modeled EQD2 is 35 Gy, much lower than that of 49.6 Gy from LQ model. EQD2 of 7.4 Gy × 5 to a radioresistant DU145 prostate cancer and to a radiosensitive CP3 prostate cancer are 56.4 and 162.9 Gy, respectively. Both are significantly different from that of 77 Gy from the LQ model using α/β = 3 Gy. Certainly, our model‐based calculations depend on the shapes of individual cell survival curves. For example, hyper‐fractionated procedures using BID is better used for tumor cells with an invert shoulder or negative curvature of n < 1. Our Eq. (2) predicts that EDQ2 depends on the fractional dose D, fraction number N, and the parameters of n and D_o_. However, LQ model estimates EQD2 using the same α and α/β ratio for 2‐Gy and high fractional doses which do not agree with in‐vivo and in‐vitro survival curves of tumor cell lines presented in Figs. 1, 2, 3, 4, 5, 6. Using LQ model of Ln(S) = αBED, EQD2 calculation should not only use clinical α/β ratio but also α value that varies with the fractional dose.

### 3.3 Fractional dose selection

**Fig. 7:**
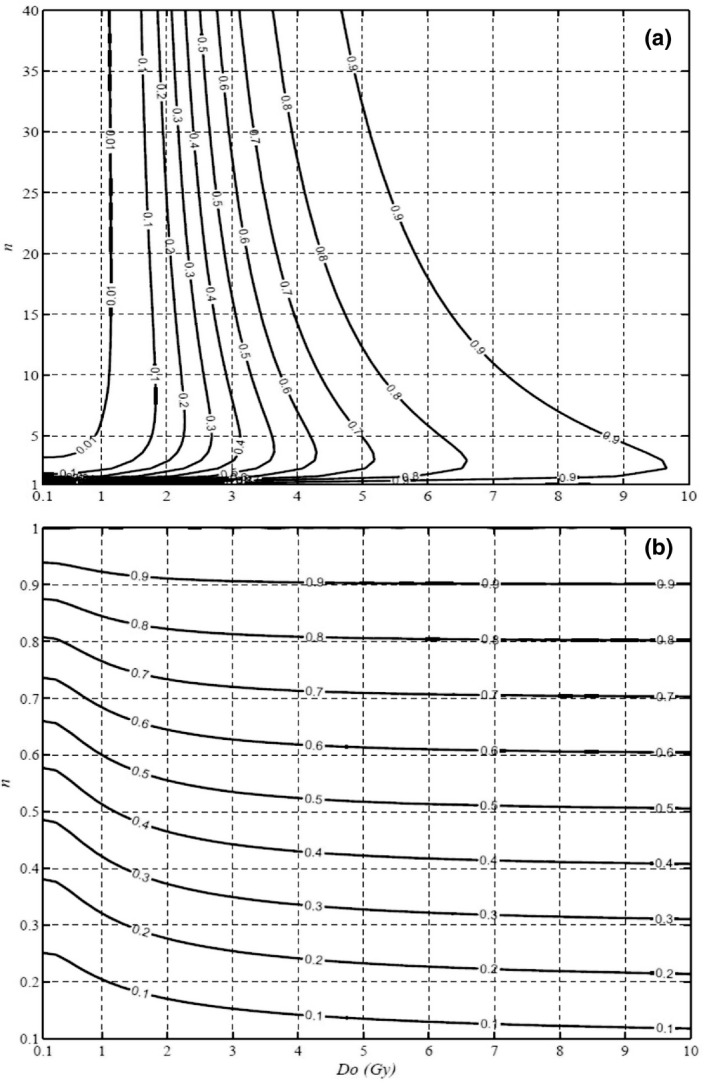
(a) Isocontours of S_10Gy_/(S_2Gy_)^5^ to show the tumor cell survival ratio for having 10 Gy delivered in one fraction as compared to the 10 Gy delivered in 2‐Gy 5‐fractions. The value increases rapidly with the value of *D_o_*. Thus, in favor of large fraction dose for cancer cells with smaller D_o_. (b) Isocontours of (S_1_._2Gy_)^2/1.2^/S_2Gy_ to present the cell survival ratio by hyperfractional BID of 1.2‐Gy fractions as compared to deliver the same dose in 2‐Gy fractions. The value increases rapidly with the value of n in favor of smaller n.

The benefits of a desired fractional dose are demonstrated by Figs. 7(a) and 7(b), presenting the ratios of S_10Gy_/(S_2Gy_)^5^ for the hypofractionated 10‐Gy fractions and (S_1.2Gy_)^2/1.2^/S_2Gy_ for the hyperfractionated 1.2‐Gy fractions in the D_o_ and n plane, respectively. The lower the isocontour value, the higher tumor control is, by delivering the same physical dose in comparison with the 2‐Gy fractions. Fig. 7(a) illustrates that tumor control in SBRT/SRT at 10‐Gy fractions rapidly decreases with the value of D_o_ but has a peak value across the value of n at $\partial \left[S_{10Gy}/{\left(S_{2Gy}\right)}^{5}\right]/\partial n=0$ for a given *D_o_* (>2 Gy). This is important since x‐ray survival curves of many cell lines have the value of n ranging from 1 to 5 and we should pay attention to both D_o_ and n in design of a radiotherapy scheme. Low S_10Gy_/(S_2Gy_)^5^ for large fractional doses in SBRT is in favor of control of tumors with small D_o_ and n > 1. But for tumor cell lines with n < 1 shown in Fig. 7(b), the tumor control rate in hyperfractionated radiotherapy of 1.2 Gy per fraction rapidly decreases with the value of n and changes less with *D_o_*, in favor of smaller n and smaller D_o_. Both Figs 7(a) and 7(b) have the highest value of 1 located along the line of n = 1. Thus, the α‐particle irradiation of the breast cancer with n = 1 has no difference among different fractional doses and it may be an advantage for taking continuously internal irradiation in theranostics using Bi‐213 alpha‐particle radiation. Certainly, the normal tissue complication should be considered in order to increase the therapeutic ratios. Due to large variations of dose and dose distribution to OARs in different treatment plans and delivery techniques, normal tissue complication requires further studies. The values of D_o_ and n along the radiosensitivity (or MID) of the tumor and normal tissue cells are essential in optimal selection of fractional doses.

## 4 DISCUSSION

This study has validated a newly proposed UMA model that can fit available survival curves of human tumor cell lines over the entire dose range with R^2^ > 0.97 and accurate predictions within the experimental uncertainties. A unified formula using only two dose‐independent parameters and a single term in a linear form of the physical dose has enabled us to reliably estimate the EDQ2 based on experimentally measured survival curves of tumor cell lines. To compare with the LQ model, we have fitted the same cell survival data by using the second order polynomial with zero intercept for LQ formula of $Ln\left(S\right)=‐\alpha D‐\beta D^{2}.$ We have obtained very good fitting with R^2^ > 0.98 for all curves but the bending of fitted curves beyond the measured dose range is of great concern. For example, LQ‐modeling of OMM2‐2 cell survival curve in Fig. 3(b) has results of R^2^ = 1.00, α = 0.497 Gy^−1^, β = 0.0827 Gy^−2^, α/β = 6.01 Gy and EDQ2 of 65 Gy in 20 Gy SRS which significantly differs from our model result of 37.4 Gy. This is due to the low α/β ratio determined directly from the survival curve in a low‐dose range. Similarly, the LQ modeling of the lung cancer cell survival curves in Fig. 4 gives results of R^2^ > 0.98, α ∈ (0.07, 0.15) Gy^−1^, β ∈ (0.016 0.069) Gy^−2^ and relatively low values of α/β ∈ (1.5, 7.2) Gy. By fitting the DU145 and CP3 cell survival curves in Fig. 5(b) and selecting dose ranges from 0 to 10 Gy and from 0 to 14 Gy, LQ modeling values of α/β ratio change from 2.14 to 4.26 for CP3 cells and from 14.0 to 18.4 Gy for the DU145 cells, respectively. Such a change greatly affects the EQD2 from the LQ model that has been routinely used for treatment decision and dose‐response assessment in SBRT, SRS, and HDRB. Our own clinical observations indicate that EQD2 from large fractional doses differ from the standard LQ model. For instance, an unexpected 13% relapse of 18 to 20 Gy SRS of over two hundreds of malignant brain metastases and primary tumors cannot be explained by LQ modelled EQD2 of 50 Gy for α/β = 10 Gy or 65 Gy for α/β = 6 Gy. Regarding a clinical question of tumor control by switching SRS to three 8‐Gy fractions SRT of brain metastases, the LQ model predicts EQD2 = 36 Gy for α/β = 10 Gy, a 28% reduction from 50 Gy of the single 20 Gy SRS. However, our model predicts EQD2 from 26.6 to 46.3 Gy for the modeled tumor cell lines with an average EQD2 of 37.9 Gy, a slight increase of EQD2 of 36.7 Gy for 20 Gy SRS. Thus, the 8 Gy × 3 SRT should have a similar tumor control as that of 20 Gy SRS. Perhaps, the selection of fractionated SRT might be in favor of decreasing EQD2 to the surrounding normal tissues based on the rapid dose drop off in OARs and fast damage repairing than that of the tumor cells. Such benefit has been observed for hearing preservation in SRT of hundreds of acoustic neuromas^34^ but the early 5 Gy × 5 scheme might have decreased EDQ2 to the benign tumors. Using the UMA model for tumor and normal tissue cell lines could provide us a theoretical analysis of the SRS/SRT of brain tumors.

In the last decade, high local control rate of early stage lung cancer treated with SBRT have been experienced by many institutions.^35^, ^36^ High EDQ2 from our UMA model and other modified LQ models^33^ might provide some explanations. EQD2 for 7 Gy × 5 HDRB of the cervical and endometrial cancer and EQD2 for 7.4 Gy × 5 SBRT of the prostate cancer decrease with D_o_ and increase slightly with n similar to the contours in Fig. 7(a). Our model calculated EQD2 for hyperfractionated radiotherapy of the tumors with inverted shoulders mainly depend on the extrapolated number n, either higher or lower than that of LQ model prediction for cells with n = 0.2 or n = 0.9, respectively. This indicates that the benefit for the selection of continuous hyperfractionated accelerated radiotherapy (CHAR) regimen depends on the characteristics of the cell survival curves. The UMA model‐prediction in Fig. 7(b) tells us which tumor cell line may benefit the most from a CHART regimen. Thus, the proposed UMA model is useful in the design and evaluation of any new fractionated radiation therapy schemes.

Our UMA model, as any new models, is not matured yet.^37^ The model has not included many factors, such as dose rate effect, cell repopulation and regroup, tumor regrowth with treatment delay, and synergistic effects for combination with other treatment modalities. Certainly, one can add more parameters and functions to improve the fitting of survival curves and address the radiation damage repairing, cell repopulation, and redistribution during a radiotherapy course. In fact, many cell survival curves have been measured with the effects of damage repairing, under hypoxic/aerobic conditions, contact, blood circulation changes such as pre‐irradiation of the medium or capillary blood vessel damages as well as altering cell phases or multiple populations of cells during in‐vivo and in‐vitro observations. If there are data from split dose experiments to simulate the current SBRT or HDRB procedures, we can check the temporal effects by remodeling the survival curves with different dose schemes. A generalized LQ model^5^ adds a modification function to the β parameter of LQ model to deal with dose rate or damage repairing for fractionated treatments but cannot explain the α and β changes with dose level in modeling the cell survival curves in comparison with the UMA modeling of the measured cell survival curves using the same dose rate but different dose levels. If the model works for observed cell survival curves in various conditions particularly for solid tumors under hypoxic, contacted, and/or pre‐irradiation conditions during the course of radiation therapy, the prediction should be applicable to clinics with similar situations. Thus, the personalized radiation therapy scheme could be achieved with patient‐specific tumor and tissue cell survival curves.

It is important to consider some mixed radiations in clinical situations such as proton therapy with tumor cells irradiated with shooting through as low LET irradiation and at the Bragg peaks as high LET irradiation. Most recently, Pfuhl *et al*.^38^ have proposed a local effect model for prediction of cell survival irradiated by mixed radiation based on the assumption that the same spatial DNA double‐strand break (DSB) distribution in the cell nucleus leads to the same effects, independent of the radiation quality. Our unified formula applicable for all types of ionization radiation over the entire dose range indicates a possible common mechanism of cell killing among various ionization radiations with their determinable MID = nLn(n)D_o_/(n‐1) — a single parameter for the description of the cell response to the radiation dose.

Better understanding of the molecular pathways of radiation‐induced cell death with multi‐activations is even more important in the design of combination therapy with the increasing use of novel agents in chemo or immune therapy.^12^ A change of biological model from a low dose of 2‐Gy fractions to a high dose of 10‐Gy fractions as does by the USC model has brought considerations for the possibility of different cell killing mechanisms in radiation therapy. Clinically observed double median survival for oligometastatic diseases^39^ and more than double the response rate to immunotherapy^40^ based on EQD2 predictions of the LQ model have led to the hypothesis that the biology of tumor response to irradiation is different when a high dose per fraction is given. Our new model has indicated that the improved local control seen in SBRT might be the results of an EQD2 much higher than the EQD2 estimated with the LQ model and there is no need of different mechanisms among different dose ranges. We have also found that some cell survival curves for combination of radiotherapy with chemotherapy or immunotherapy as well as hyperthermia^23^, ^25^ could mostly be fitted well with the unified formula (to be presented with our other report). Such a unified formula representing the same mechanism through the entire dose range or combination of multiple treatment agents (including radiation) allows us to describe the dose response without changing of the mechanisms or dose prescription.

## 5 CONCLUSIONS

The feasibility of the proposed UMA model has been validated through the fitting of survival curves of many tumor cell lines available to us. The capability of modeling survival curves of in‐vivo and in‐vitro human cell lines over the entire dose range within their experimental errors provides us a new way to calculate EQD2 of SRS, SBRT, HDRB and even hyperfractionated radiation therapy courses. In comparison with the current LQ model estimations, this study has found EQD2 that is lower for intracranial SRS but higher for SBRT of lung cancer using parameters extracted from some preclinical cell survival curves. Most importantly, the unified formula has resolved the catastrophe of the traditional LQ and MT models and it theoretically indicates a common mechanism of cell killings from ionization radiation and possibly from other agents at all dose levels.

## Conflict of Interests

The authors have no other relevant conflict of interest to disclose.
